# Understanding Sensor Cities: Insights from Technology Giant Company Driven Smart Urbanism Practices

**DOI:** 10.3390/s20164391

**Published:** 2020-08-06

**Authors:** Gaspare D’Amico, Pasqua L’Abbate, Wenjie Liao, Tan Yigitcanlar, Giuseppe Ioppolo

**Affiliations:** 1 Department of Economics, University of Messina, Via dei Verdi, 75, 98122 Messina, Italy; 2 Department of Civil, Environmental, Building Engineering and Chemistry, Polytechnic of Bari, 70125 Bari, BA, Italy; p.labbate@tiscali.it; 3 Institute of New Energy and Low-Carbon Technology, Sichuan University, Chengdu 610065, China; wenjieliao@outlook.com; 4 School of Built Environment, Queensland University of Technology, 2 George Street, Brisbane, QLD 4000, Australia; tan.yigitcanlar@qut.edu.au

**Keywords:** sensor city, City 4.0, sustainable urban development, smart city, smart urbanism, smart governance, disruptive urban transition, Internet-of-Things (IoT), technology giants, sensors

## Abstract

The data-driven approach to sustainable urban development is becoming increasingly popular among the cities across the world. This is due to cities’ attention in supporting smart and sustainable urbanism practices. In an era of digitalization of urban services and processes, which is upon us, platform urbanism is becoming a fundamental tool to support smart urban governance, and helping in the formation of a new version of cities—i.e., City 4.0. This new version utilizes urban dashboards and platforms in its operations and management tasks of its complex urban metabolism. These intelligent systems help in maintaining the robustness of our cities, integrating various sensors (e.g., internet-of-things) and big data analysis technologies (e.g., artificial intelligence) with the aim of optimizing urban infrastructures and services (e.g., water, waste, energy), and turning the urban system into a smart one. The study generates insights from the sensor city best practices by placing some of renowned projects, implemented by Huawei, Cisco, Google, Ericsson, Microsoft, and Alibaba, under the microscope. The investigation findings reveal that the sensor city approach: (a) Has the potential to increase the smartness and sustainability level of cities; (b) Manages to engage citizens and companies in the process of planning, monitoring and analyzing urban processes; (c) Raises awareness on the local environmental, social and economic issues, and; (d) Provides a novel city blueprint for urban administrators, managers and planners. Nonetheless, the use of advanced technologies—e.g., real-time monitoring stations, cloud computing, surveillance cameras—poses a multitude of challenges related to: (a) Quality of the data used; (b) Level of protection of traditional and cybernetic urban security; (c) Necessary integration between the various urban infrastructure, and; (d) Ability to transform feedback from stakeholders into innovative urban policies.

## 1. Introduction

Today, cities are at the forefront of increasing urbanization and digitalization pressures [1,2,3], where they play a crucial role in supporting the transition towards a sustainable and smart urbanism practice [4,5,6]. The current urban context is associated with numerous economic, social and environmental issues, such as waste management [7,8,9,10], energy efficiency [11,12,13], renewable energy sources [14,15], water management [16], social, cultural, and health aspects [17,18,19,20,21,22], material flows [23], biodiversity [24], transport [25,26], land use optimization [27], air and noise pollution prevention [28,29,30], infrastructure mishaps [31], economic growth [32]. Policymakers, thus, need a paradigm shift, developing innovative, sustainable, and intelligent solutions to optimize urban processes and improve citizens’ quality of life and sustainability of the city [33,34,35,36].

In recent years, the notion of ‘sensor city’ has emerged as a response to the future challenges of growing urbanization and datafication [37,38]. This new version of the city—i.e., City 4.0,—(thanks to the urban dashboards and platforms integrates Internet-of-Things (IoT) infrastructure [39], sensors [40], real-time monitoring stations [41,42], digital cameras [43], actuators [44], real-time tracking systems [45,46], big data analytical techniques [47,48,49], information and communication technologies (ICTs) [50,51], cloud computing [52], smart grid [53,54], artificial intelligence (AI) [55,56,57], autonomous shuttles [58], and other digital appliances with physical objects that characterize urban context) improves the efficiency of resources usage.

Specifically, city dashboards accommodate visual/graphical and dynamic analysis suite capable of holistically combining urban infrastructures to view, integrate and communicate real-time information on performance, trends, and future urban scenarios [59,60]. These dashboards are characterized by a high degree of interactivity with users, capable of combining, filtering, querying and overlapping large amounts of urban data [61]. Indeed, urban dashboards are implemented to facilitate an understanding on major urban issues and provide stakeholders with a sense of accountability and engagement on smart urban governance activities [62,63,64].

According to the forecasts of the “World Smart Cities Spending Guide” provided by the International Data Corporation [65], the total expenditure for smart urban solutions this year alone amounted to almost USD 124 billion. This is an increase of 18.9% compared to 2019 [65]. Specifically, global cities such as Singapore, Tokyo, New York City and London occupy the top of the ranks in terms of investments in smart urban initiatives [65]. Furthermore, cities such as Toronto, Adelaide, Hamburg, Kansas City, Dallas and Stockholm (see Table A1 in Appendix A) have implemented participatory and intelligent platforms that use ICTs to connect companies, local authorities, universities, start-ups, citizens, associations, and so on in order to support the decision-making process, allowing the collection, processing, monitoring and analysis of large amounts of urban data [66,67,68]. In this sense, Bibri [34] described cities as complex networks of holistic relationships that integrate smart and sustainable solutions in order to provide a suitable context for long-term urban strategic development.

Indeed, the rapid and pervasive development of ICTs taking place all over the world is transforming cities into centers of economic, social, environment and technological development with the aim of providing increasingly efficient, sustainable and smart urban services [37,50,69,70]. In this regard, the United Nations (UN) Agenda 2030 defines ICTs as necessary tools to facilitate the transition towards sustainable development [71]. Hence, policymakers use data and information sharing systems through IoT technologies for planning, monitoring, and evaluating the performance of urban policies, and for improving transparency, active participation of citizens and awareness of urban issues [72,73,74]. For example, cities such as Singapore, Zurich, Oslo, Geneva, Copenhagen, Auckland, Melbourne, Taipei, Helsinki, Bilbao and Düsseldorf represent forward-looking cases regarding the use of ICTs in the urban area, occupying the first places in the ranking developed by the IMD World Competitiveness Center Smart City Observatory [75].

The current urban theoretical and managerial debates increasingly focus on the role of ICTs and their integration with various aspects related to sustainable urban development [76,77]. Indeed, the literature provides several synonymous for sensor city such as digital city [78,79,80], smart city [81,82,83], ubiquitous city [84,85,86], knowledge city [87,88,89], intelligent city [90,91], techno-centric city [92], creative city [93,94], sustainable city [95,96], informational city [97,98], smart sustainable city [99,100,101,102], and artificially intelligent city [57], which express the importance of ICTs in the management of the cities of future.

Nevertheless, technology alone cannot be a panacea for all urban issues related to growing urbanization [103,104,105]. In particular, cities excessively connected to IoT and big data analytical solutions have often been criticized for being too techno-centric and for underestimating social and environmental aspects [106,107,108]. At the same time, sustainable cities struggle to integrate the technological approach with the social, environmental and economic dimensions of sustainable development [102,109]. Specifically, the use of advanced techniques (e.g., real-time monitoring stations for energy consumption, location systems to guide urban traffic, cloud computing systems for sharing sensitive data between government departments, urban infrastructures such as smart bins, smart street lamps, and surveillance cameras) highlights a multitude of challenges related to the quality of the data used, level of protection of traditional and cybernetic urban security, necessary data integration between the various urban infrastructures, and ability to transform feedback from citizens and other stakeholders into innovative urban policies. Consequently, sensors and related ICT infrastructures are rapidly gaining strategic importance for sustainable and disruptive urban development [110]. They are not only enhancing in terms of technological aspects, but also social, environmental, and economic ones.

This paper aims to explore the main challenges related to sensor cities, emphasizing the opportunities and critical issues of this growing datafication of urban contexts. In this sense, an integrated and holistic framework is proposed which includes a theoretical and managerial review of the main disruptive technological applications. The study, thus, identifies and compares IoT solutions based on sensors, big data analysis and other technologies related to ICTs adopted by different cities, to manage urban development in an innovative and computerized manner. This paper generates insights from the current sensor city best practices by placing some renowned projects, implemented by Huawei, Cisco, Google, Ericsson, Microsoft, and Alibaba, under the microscope. With the objective to offer a detailed overview, the paper adopts a mixed research approach able to integrate a literature review and an in-depth analysis of several case studies. In this regard, the proposed framework provides users with greater knowledge and awareness of sensor cities’ development.

The paper is structured as follows. Section 2 introduces the mixed methodological approach used. Section 3 analytically describes the proposed framework, highlighting the technological factors such as IoT, big data analysis, AI, ICTs, real-time monitoring stations, sensors, cloud computing, digital platforms, and urban challenges that characterize sensor cities. Finally, Section 4 provides conclusions and some considerations on the contribution of sensor cities to future urban challenges.

## 2. Materials and Methods

**Figure 1:**
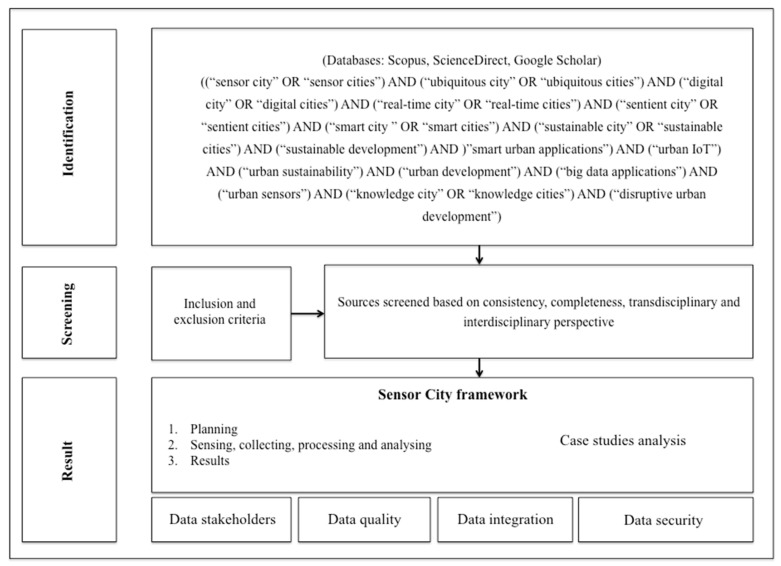
Methodological approach adopted (source: authors).

The development of this study is structured according to a mixed approach, characterize by a literature review and a detailed analysis on several case studies, in order to create a framework for policymakers that collect the forward-looking ICT urban initiatives, emphasizing a data-focused method in the assessment of urban development. The implementation of this study is carried out in different phases and in this sense the methodological approach followed is illustrated in Figure 1.

In Phase 1: Identification, the research question, keywords, and research databases are defined. Regarding the objective and the research question, the study aims to identify and analyze the main characteristics of sensor cities, highlighting the impacts of their technological applications on urban development.

The attention of the study has focused that have provided an interdisciplinary and/or transdisciplinary perspective to the development of sensor city. Specifically, these scientific disciplines include technology and innovation management, urban planning, policy, sustainable development, environmental management, data science, urban informatics, geography, urban development, strategic management, and urban statistics. Moreover, the paper—through a qualitative approach—analyzes several sensor cities considered successful examples of disruptive urban actors, capable of integrating sensor strategies with the economic, social, and environmental aspects of urban development in detail. As a result, the study includes peer-reviewed journal articles, book chapters, and conference proceedings, grey literature such as government documents, and industry technical reports. In terms of databases, ScienceDirect, Google Scholar and cities’ websites were utilized to achieve the analysis. Furthermore, the keywords searched include ((“sensor city” OR “sensor cities”) AND (“ubiquitous city” OR “ubiquitous cities”) AND (“digital city” OR “digital cities”) AND (“real-time city” OR “real-time cities”) AND (“sentient city” OR “sentient cities”) AND (“intelligent city” OR “intelligent cities”) AND (“data-driven city” OR “data-driven cities”) AND (“smart city” OR “smart cities”) AND (“sustainable city” OR “sustainable cities”) AND (“sustainable development”) AND (“smart urban applications”) AND (“urban IoT”) AND (“urban sustainability”) AND (“urban development”) AND (“big data applications”) AND (“urban sensors”) AND (“knowledge city” OR “knowledge cities”) AND (“disruptive urban development”).

In Phase 2: Screening, the review aims to provide a clear and comprehensive definition of the concept of sensor city, introducing urban dimensions (e.g., governance, economy, environment, mobility, people, and living) and technological solutions (e.g., IoT, sensors, AI, ICTs, big data analytics) necessary for its implementation. In order to further refine the research, all the selected sources were screened following a set of inclusion and exclusion criteria, in line with the objective and the research question of the study. As exclusion criteria, sources with partial information and inconsistent with the topic of the study were not included in the search.

In Phase 3: Result, the integration between literature review and case study analysis provides a detailed framework useful for developing a theoretical approach to sensor cities, underlining the holistic relationships between urban sustainability and computerization aspects. Nonetheless, it is important to consider that sensor cities taken into consideration in the case studies analysis use non-uniform terminology and lack detailed quantitative data on sensor infrastructures. In this sense, a greater understanding and awareness of urban sensing is needed to develop forward-looking projects in line with future urban challenges. This research focused on 20 sensor cities, with a high quality in social, economic, environmental and technological infrastructures. Consequently, the sample analyzed does not represent the different types of cities globally. Most cities around the world are not equipped to collect, monitor, analyze and evaluate urban performance through innovative platforms and dashboards. In sum, this review provides: (a) A clear definition of sensor city; (b) A sensor city framework; (c) A detailed analysis of several sensor cities, and; (d) Various different sensing policy and actions that currently policymakers have in place.

## 3. Results

Given the growing importance of sensor city solutions in the world, technology giant companies (e.g., Huawei, Ericsson, Microsoft, Oracle, Alibaba, Deutsche Telekom, Samsung) are involved in numerous urban technological projects in order to provide platforms, products and services in line with the necessary paradigm shift [111]. Thus, sensor cities aim to involve citizens and companies in the process of planning, monitoring, and analyzing of urban processes and raising awareness and comprehension on environmental, social, and economic issues [112,113]. Table A1 in the Appendix A presents some examples of cities equipped with real-time monitoring stations and sensors that analyze and collect urban performance.

Few [114] in his book ‘Information Dashboard Design: The Effective Visual Communication of Data’ defines the dashboard as a visual, consolidated and organized display on a single screen of the most important information needed to monitor, analyze and achieve one or more urban objectives. Similarly, Kitchin [115] explains urban dashboards as digital, physical or mixed interfaces that allow users to actively or passively interact in urban data monitoring and management in order to improve understanding of urban systems. Kitchin and McArdle [116] and Petit and Leoa [117] describe urban dashboards as platforms that use dynamic and interactive graphic interfaces, maps, 3D models, augmented reality, bar charts, and so on, to support urban decision-making with the aim of monitoring, analyzing and interpreting performance and trends of cities. Thus, the sensor city is generally accepted as a digital platform where users are completely immersed.

Nevertheless, theoretically, the ideals that characterize sensor cities can be defined as universal and homogeneous, while contexts tend to be specific and heterogeneous [118]. For example, cities have different social, economic, environmental and technological infrastructures and are governed by different political and bureaucratic systems with interests that are difficult to combine in a single urban project. Furthermore, while urban data is increasingly easy to access, technical and digital skills are difficult to find in some urban contexts around the world, in order to manage, analyze and interpret urban data.

Urban contexts using IoT and/or ICT platforms require more initial efforts due to the assembly and maintenance of the infrastructure, in order to adapt it to increasingly complex urban scenarios [119,120]. In detail, it is a matter of integrating devices that are: (a) Heterogeneous, capable of generating different types of data; (b) Powered by different energy sources, such as battery or renewable energy; (c) Dynamic and flexible, capable of analyzing constantly changing urban scenarios, and; (d) Unpredictable, in the sense that technological applications can provide conflicting results by analyzing the same data, as they use different protocols and standards.

The significant costs of designing, installing, and maintaining monitor stations and sensor technologies represent a barrier to entry for smaller cities or those located in less development regions [121,122]. The success of an urban sensor strategy depends essentially on the economic, social and environmental characteristics of the urban context taken into consideration together with organizational, ethical and transdisciplinary factors of the actors involved [118].

**Figure 2:**
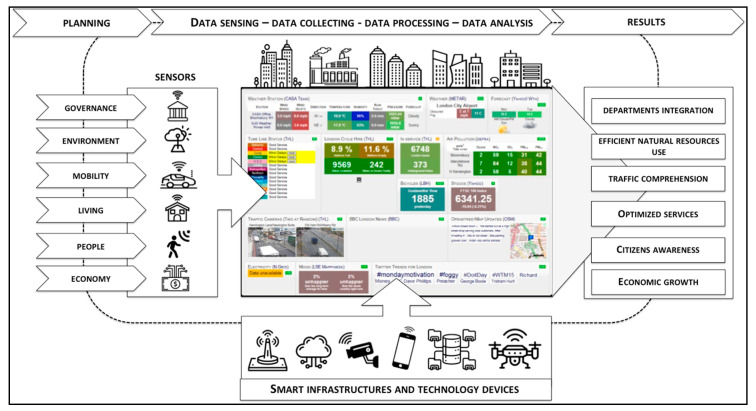
Sensor city framework (source: authors).

The sensor city framework is illustrated in Figure 2. In detail, the operational phases, integrated in a holistic perspective of the urban context, are divided into planning, sensing, collecting, processing and analysis of urban data and results.

In Phase 1, or planning, the role of ICTs is highlighted, which allows citizens to participate in the decision-making process and to enhance systemic collaboration between stakeholders involved, contributing significantly to greater comprehension, transparency and accountability [123,124,125]. Hence, planning activities through smart solutions permits a holistic and integrated approach of the various urban dimensions (e.g., governance, economy, environment, mobility, living, and people), reducing costs and time of the bureaucratic collaborations between departments and improving quality and efficiency of urban services [126]. Nonetheless, most cities do not work like companies (e.g., IBM, Cisco, Google). They tend to be disorganized, e.g., departments do not collaborate on solutions [127]. On this point, Cugurullo [128] elaborates that urban contexts promoted as examples of integrated and holistic urban planning are often fragmented and disconnected, characterized by several incompatible components.

The Digital Single Market strategy adopted by the European Commission (EC) represents one of the fundamental pillars of the policies for creating a digital single environment [129]. Indeed, the strategy aims to ensure a better, secure and uniform access for citizens to digital networks in order to improve technological knowledge and encourage greater social inclusion.

The governance dimension highlights the spread of social media (e.g., Facebook, Twitter, Instagram) and other data sharing platforms that allow increasingly integrated and structured communications between stakeholders [130]. Thus, by adopting a bottom-up approach, these feedback-generation, sharing and management tools transform citizens, companies, local authorities, and so on, into active participants in the governance of sensor cities [131,132,133].

The environmental dimension involves various aspects of urban context such as:

▪ Waste management, capable of providing real-time type and quantity of waste via cloud solutions, to optimize time and resources [10,134]. With the development of new technology devices, electronic waste (e-waste) represents an environmental and health challenge due to its difficult disposal and potential impacts on the environment.
▪ Energy efficiency, which refers to all technologies able to reduce energy consumption. For example, street lighting networks equipped with sensors and public and private buildings equipped with real-time consumption monitoring systems represent the forward-looking urban infrastructures needed to manage urban activities efficiently [12,13,135].
▪ Renewable energy sources, such as solar, wind, thermal and biogas, which represent a sustainable and efficient alternative to fossil fuels, capable of guaranteeing a stabilization of energy prices and a sustainable source of energy supply for urban processes (e.g., public transport, heating of public and private buildings) [14,15].
▪ Water management, which includes solutions capable of providing real-time data on the consumption and quality of water distribution system, fundamental for the sustainability and efficiency of the urban system [16].
▪ Material flows, those underline the exchange and transformation of resources (e.g., raw materials, by-products, waste) between various interested actors [23].
▪ Biodiversity conservation, which includes revitalization actions for abandoned urban and industrial areas and the safeguarding of green and urban spaces [24].
▪ Land use optimization, as support for urban agriculture and infrastructure [27].
▪ Air and noise pollution prevention, to reduce pollutant emissions through ICTs, sensors, and real-time monitoring stations, capable of providing high-definition videos and images to check environmental quality [30].

The mobility dimension includes, amongst others, smart and sustainable public transport system, availability of urban infrastructure suitable for autonomous vehicles, car sharing stations for electric vehicles and ICTs relating to traffic and road monitoring [25,136,137,138].

The aspects related to the living dimension include actions to improve the use of cultural and entertainment facilities (e.g., libraries, museums, schools, public parks, sport facilities) [20].

The people dimension includes policies aimed at promoting a ‘sustainable’ community, improving digital literacy, providing various assistance programs for citizens with special needs and a quality healthcare system, ensuring gender equality in terms of pay and office positions, safe and healthy work environments, and so on [139].

The economic dimension refers to the ability of the urban context to favor a path of growth through technological innovation, entrepreneurship and sustainability, attracting the most innovative companies, start-ups and talents, and capable of promoting a digitalized and collaborative development [32].

**Figure 3:**
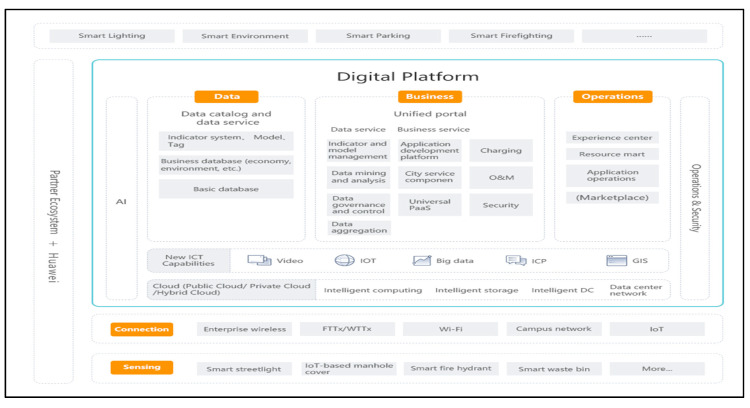
Huawei Digital Platform, derived from [141].

In Phase 2, with the objective of sensing, collecting, processing and analyzing urban data, the Chinese telecom company Huawei, for example, has developed ‘Smart City Solution’, a platform used in over 160 cities in 40 countries in Asia and Europe, capable of analyzing large volumes of real-time urban data and providing policymakers a method of predictive analysis of future urban scenarios (Figure 3). Specifically, Lanzhou New Area represents the first new state-level development area in northwest China which, through the support of Huawei’s network, was able to build the nation’s first governmental IoT and wireless sensors network that integrates both broadband and narrowband communications, integrating 31 departments (e.g., public safety, finance, energy, transport, healthcare, education) with 45 eLTE stations. The program aims to improve the quality of life by optimizing urban resources in a smart and sustainable manner [140].

**Figure 4:**
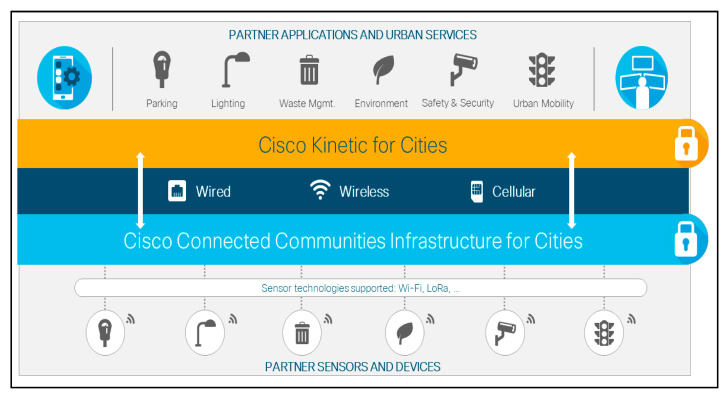
The Cisco Smart + Connected Digital Platform, derived from [143].

Similarly, Cisco has developed the ‘Cisco Kinetic for Cities’ framework, an open and easy-to-use urban data sharing platform that combines data provided by sensors, applications and other third-party devices to create a dynamic sensor city infrastructure, encouraging the exchange of innovative urban initiatives between policymakers, companies and citizens (Figure 4) [142].

**Figure 5:**
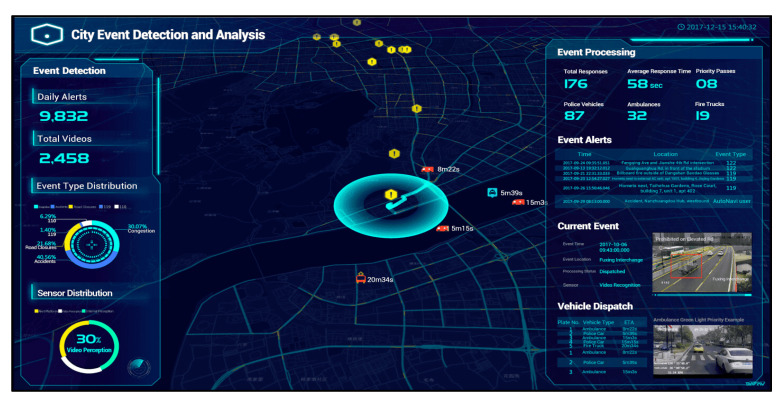
Alibaba City Event detection and smart processing, derived from [145].

At the same time, Alibaba has developed the ‘City Brain’ program that has also become a useful tool for city managers, providing a holistic dashboard that can improve the perception of urban data and real-time processing capacity [144]. Figure 5 shows the real-time detection and analysis platform for city events. By integrating the data from the surveillance rooms, it is possible to coordinate traffic lights to give priority passage to response vehicles (e.g., police, fire-fighters, rescue and other vehicles) in case of emergency. Likewise, the program has been launched in several Chinese cities such as Hangzhou, Shanghai, Chongqing, Suzhou, Haikou, Beijing, Chengdu, Quzhou, and Jiaxing. Projects as CityBrain highlight the continuous interaction between local authorities and technology companies in managing urban governance. In fact, the Chinese platforms are largely owned by national technology companies [60].

Barcelona has changed its sensor city approach from top-down to bottom-up, involving its citizens in participating in innovative urban projects. For example, the Smart Citizen Kit is a dashboard that collects data on the environmental such as air composition, temperature, light intensity, sound levels and humidity through sensors and ICTs [146]. The data collected in real-time are sent via Wi-Fi to an open data platform and are used to create maps that display environmental conditions, equipping public and private stakeholders with urban data in order to develop and/or improve services for citizens [147]. Telefonica—through the Valencia Smart City Project—aims to transform the city of Valencia into an intelligent ecosystem and fully connected via 350 sensors, allowing the management of public resources through a single ICT platform and improving several urban areas such as transport, energy, efficiency and environmental services. The city of Santander has installed around 20,000 parking sensors in the streets, introducing intelligent waste containers capable of monitoring and measuring air pollution, rainfall, and traffic density. Through the integration and interpretation of the corresponding data, the Santander municipal administration optimizes waste truck routes to save staff and fuel costs, or controls the irrigation of city parks to save water [148].

**Figure 6:**
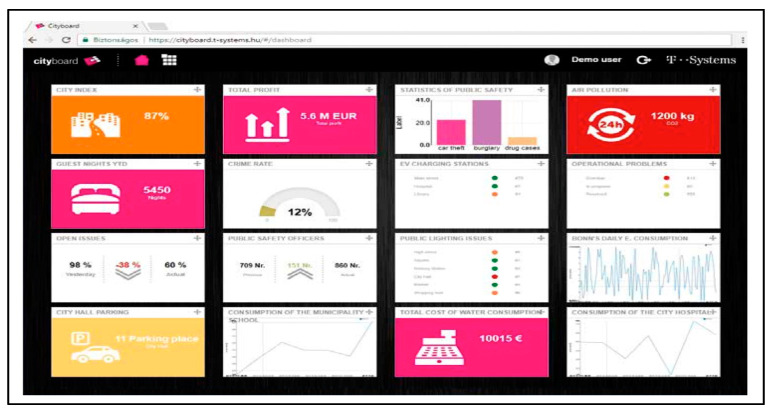
Smart City Dashboard developed by Deutsche Telekom for Hungary subsidiary, derived from [149].

Deutsche Telekom have implemented similar dashboard, equipping urban physical object (e.g., street lamps, bins, parking lots, traffic lights) with software, sensors and connectivity systems integrated into a shared network. In this sense, the collection, monitoring, analysis and interpretation of urban data allows the implementation of innovative service and business models (Figure 6).

In 2018, Deutsche Telekom and the city of Hamburg implemented around 11,000 parking spaces equipped with sensors that can provide the current availability status to users via the app [150]. The local authorities of Gelsenkirchen (Germany) have decided to collaborate with Huawei and GELSEN-NET in order to implement a new open and shared urban governance system, developing an ICT infrastructure capable of integrating key data to improve the efficiency of the public services and the accuracy of the decision-making process. In particular, the ‘Safe City’ program uses Huawei’s extensive wired and wireless broadband network and the IoT that connects industrial parks, hospitals, schools, pedestrian areas and urban centers, creating a sustainable ICT ecosystem. As part of the T-City project in Friedrichshafen (Germany), Deutsche Telekom has partnered with Alcatel-Lucent to provide hardware solutions and network devices for T-City. Specifically, Deutsche Telekom has developed over 40 pilot projects in several categories such as mobility, research, tourism, culture, health, and employment.

The LuxTurrim5G ecosystem in Espoo (Finland) aims to transform the Kera area into a sustainable and digitized urban neighborhood, involving Nokia and several partners in order to create a multitude of urban digital services. The LuxTurrim5G platform is able to collect, store, manage and share large amounts of urban data and solutions in a safe and efficient way between local authorities, companies and citizens. Specifically, the network developed by Nokia includes, among other things, over 50 Wi-Fi devices, 75 cameras, 49 different sensors that monitor air quality, climate, temperature, road surface conditions as well as CO_2_ levels, 9 radar devices, 7 information screens, a charging station for electric vehicles and a charging and landing station for drones.

Oracle through a partnership with the Indian state of Maharashtra has implemented a platform to design, develop and analyze government-to-citizen and smart-business government services across the state. Maharashtra also aims to connect all its 113 million residents via fiber, including those in the more than 300 cities of Maharashtra and its 29,000 villages. Dallas has chosen to collaborate with Ericsson to implement an advanced traffic management system through a dashboard capable of monitoring, managing, analyzing and aggregating different data in real-time from traffic sensors and cameras to dynamically control the traffic lights. The goal of the city is to have an intuitive and shared analysis tool capable of integrating data from the various departments and agencies.

**Figure 7:**
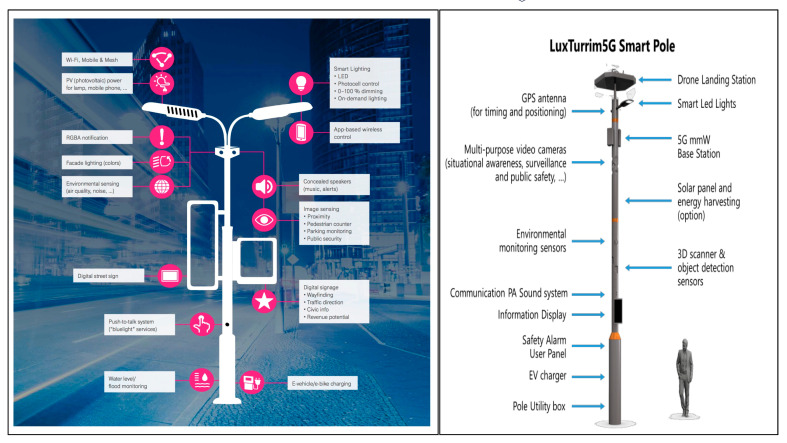
Smart Street Lighting models developed by Deutsche Telekom and Nokia, derived from [151,152].

According to the report ‘The future of street lighting’, street lighting represents the main part (about 40%) of a city’s overall electricity costs. In this sense, the replacement of conventional lamps with LED bulbs can reduce energy consumption by up to 80% when using a centralized management system. For example, intelligent lighting platform of Cisco and Sensity in Kansas transforms each lamppost into a sensor connected to a broadband wireless network, creating a true interconnected lighting network capable of collecting real-time data such as intelligent parking systems, electricity consumption, or air quality level. In this sense, the smart pole developed by Deutsche Telekom and Nokia represents a paradigm shift in the use of lighting poles as it goes beyond simple urban lighting and constitutes an essential component of the infrastructure of sensor cities (Figure 7).

The Hamburg Port Authority (HPA) through Cisco systems has developed sensors that monitor the use of resources (e.g., trucks, cranes, means of transport, ships) and infrastructures (e.g., roads, parking lots, storage warehouses) in one of the busiest and most important ports in Europe. In this sense, modernization through IoT technological innovation allows the Port Road Management Center to plan future investments in the traffic infrastructure, optimizing the flow of traffic and minimizing the externalities of the port on the inhabitants of the city.

Ericsson aims to make Stockholm the smartest city in the world by 2040. With the water Monitoring Network project, Ericsson has the ability to design and implement a real-time water quality-monitoring network using an IoT sensor system located in the Stockholm water system. In addition, big data analytics are used that can analyze the data produced by the sensors and provide more information on changes in water quality such as pH and temperature.

In many cases, small towns do not need sophisticated public transportation solutions. In this sense, the challenge is to combine the departure time of buses, subways, railways, and so on, with data from traffic jams and road works on highways, showing the best alternative connections. As a partner of the Kooperation Östliches Ruhrgebiet, Deutsche Telekom helps to connect local public transport in the German state of North Rhine-Westphalia. In this regard, Deutsche Telekom’s strategy allows not only meeting the needs of passengers in terms of both transparency in information (e.g., timetables, delays) and efficiency, sharing the same platform for multiple tasks. In Croatia, Deutsche Telekom’s subsidiary, Hrvatski Telekom, has developed an electric vehicle charging network of 145 charging points in 101 charging stations in 70 cities. The project also integrates an ICT infrastructure that helps users to book and pay for vehicles top-ups and receive real-time availability information.

The final phase concerning the results explaining the decisive role of sensor solutions in terms of implementation of innovative urban processes (e.g., efficient use of natural resources, cross departmental integration, real-monitoring of traffic congestion and energy consumption, smart management of infrastructures), in order to strategically improve the contribution of sensor cities to disruptive urban development. Consequently, the optimization of Quality of Experience (QoE) and Quality of Services (QoS) has become a crucial aspect in the implementation of urban services and processes [153]. Particularly, QoE represents an evaluation of human experience when interacting with technology solutions and stakeholders in a given context. Therefore, a detailed analysis of the QoE should consider several actors interacting with each other at different levels (environmental, social, economic, technological) and with different goals. To do this, it is necessary to define the main interactions between citizens, companies, local authorities, social organizations, and so on. In sum, some QoE aspects related to sensor city are listed in Table 1.

In this regard, the challenge for policymakers will be the widespread use of several tools, such as devices, platforms, algorithms, and networks, to access, share and integrate the largest number and type of urban data. The big data evolution process requires significant data gathering, highly specialized personnel, and infrastructures owned by different municipalities, agencies, corporations, and private companies [5]. This attention of ICT leads urbanist mark Swilling to describe the sensor city as a form of algorithmic urbanism [154]. In this sense, concepts, such as big data analysis, ICT, sensors, real-time monitoring systems, smart infrastructures, IoT, represent necessary but not sufficient solutions [105,115,155]. The reconfiguration of the urban system expresses the need to integrate the environmental, social, economic dimensions of smart technologies into an integrated, efficient, and computerized urban context [6,49,156,157].

## 4. Discussion

The perspective of implementing sensor cities based on IoT and on different urban technologies that permit the collection, monitoring, analysis and integration of large amounts of urban data has developed new opportunities for policymakers regarding the ability to plan, combine and evaluate social, environmental and economic aspects in a holistic manner [34,158,159].

**Figure 8:**
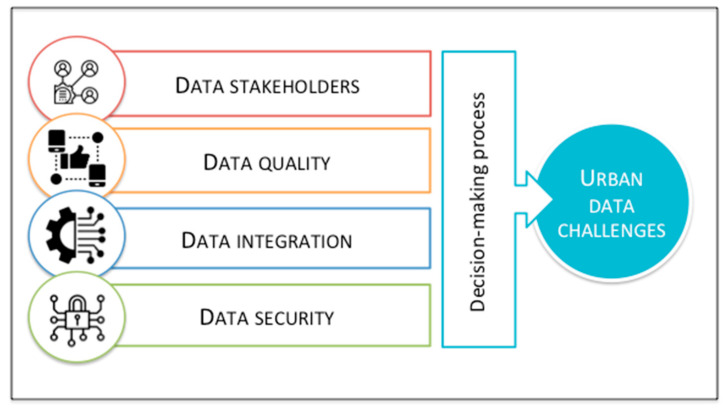
Future urban data challenges (source: authors).

The expansion of urban technologies has stimulated local authorities, technology companies, start-ups, citizens, municipal companies, and other actors involved, to develop increasingly innovative and sophisticated projects, devices, platforms, algorithms, systems, initiatives, and so on, especially in technologically and ecologically developed cities. Consequently, the use of advanced techniques such as real-time monitoring stations for energy consumption, location systems to guide urban traffic, cloud computing systems for sharing sensitive data between government departments, urban infrastructures such as smart bins, smart street lamps, and surveillance cameras, have great potential in transforming the way we understand and evaluate the urban context and, at the same time, highlight a multitude of challenges related to the quality of the data used, the level of protection of traditional and cybernetic urban security, the necessary data integration between the various urban infrastructures and the ability to transform feedback from citizens and other stakeholders into innovative urban policies (Figure 8). In that perspective, sensor cities represent a sophisticated paradigm shift in the concept of the technical-urban context, necessary for a transition towards disruptive urban development [160,161,162].

The first challenge refers to the development of innovative urban policies in line with the needs of citizens, local authorities, companies, organizations, and so on. Hence, to use a significant amount of urban data according to the needs of the stakeholders involved, it is necessary to identify the relevant information and understand its consistency and validity. For example, in a survey, citizens of the well-known German tourist destination, Heidelberg, identified traffic as the most urgent issue to be solved. In this sense, the efforts of local policymakers have been focused on how to improve urban mobility. Thus, the identification, collection and processing of information provided by the actors involved are crucial in the development of urban sensor projects [163,164,165].

The second challenge concerns the need to guarantee a certain level of data quality in order to develop efficient and innovative urban policies. For example, data collection sensors can produce incorrect, partial or missing values as they use different standards or protocols, generating inconsistencies and differences between the data. For example, CityPulse described by Puiu et al. [166] is a real-time monitoring framework supported by the European Union (EU) that processes, integrates and adapts uncertain and incomplete data through quality of information techniques in order to develop reliable information capable of satisfying user requests. In this regard, the urban project represents a practical model of how to move from vertically to horizontally interconnected services [167].

Cities with concentrated IoT and sensor networks may provide unreliable communications due to incorrect data transmission. Specifically, the incorrect transmission of urban data does not only require time, but also retransmits the information flow, which negatively affects the quality of the data [168]. As a result, the quality of data and the technologies to overcome problems of inconsistency, partiality, and unreliability should be considered in the development and implementation of sensor city projects [169,170].

The third challenge concerns the integration of different types of data, collected, processed and analyzed by different institutions, companies and/or independent authorities. One of the main tasks of public decision-makers, and urban managers, planners, and policymakers is to implement infrastructures, models, and networks capable of connecting data deriving from different urban sectors, promoting better communication between the various stakeholders involved [171]. For example, the dashboard implemented by Alibaba, illustrated in Figure 5, requires an information flow and a systemic communication between government, security and emergency service institutions. Hence, the holistic combination of data is necessary to plan and better understand the potential of sensor cities.

The fourth challenge involves the security of sensitive data. In this regard, ensuring maximum privacy protection is indispensable for developing urban policies based on sensors, video surveillance cameras, monitoring stations, cloud computing, and so on. Data security issues not only have isolated effects, but also often affect all the urban dimensions (analyzed in the previous section). Thus, data security is treated as a fundamental issue in the management of sensor cities [172,173,174]. Through the collection and use of large amounts of data and technological solutions that analyze human behavior, it is possible to influence not only the fight against crime through sensors that analyze movements or facial expressions, but also urban governance in terms of planning and the attractiveness of the city [175,176,177]. Nonetheless, the corresponding interventions are particularly controversial in terms of privacy, emphasizing ethical requirements in the urban safety planning process [178,179]. Therefore, integrating the problems related to traditional and cybernetic urban security into the planning and implementation projects of sensor cities is necessary to guarantee safe and digitalized urban development [180,181].

The challenges explained in this section are related because the collection, processing and analysis of data through IoT solutions, sensors and big data analysis refer to interdependent urban dimensions (e.g., governance, environment, mobility, economy, life and people). Nevertheless, this perspective requires a change in the structural paradigm of technical-scientific skills, greater organizational flexibility of government institutions and a more aware and involved citizenship in the urban administration.

## 5. Conclusions

Sensor and IoT technologies are acquiring ever-increasing importance in the techno-spatial context by collecting, processing, analyzing, and integrating large amount of data to improve the healthy functioning of our cities. In this regard, the importance of the corresponding urban assessment tools is evidenced by the vast investment made by technology giants, such as Cisco, Google, Microsoft, Ericsson, Alibaba, Oracle, in prototype sensor cities. These initiatives are helping cities, such as Toronto, Valencia, Dallas, Singapore, Kansas City, Hamburg, Shenzhen, Adelaide, Dublin, integrate IoT and related applications for big data, and actively engaging in city sensor development. The sensing city model allows policymakers to benefit from big data and urban analytics to improve urban policies, services, and operations.

The purpose of this paper is to explore and integrate different sensor cities taken as case studies consistent with the research demand and to discuss technological solutions (e.g., sensors, devices, IoT, AI, platforms, digital infrastructures, computer models, ICTs), emphasizing the economic, social and environmental benefits of their practical application. Hence, the technological applications enabled by the IoT and ICTs in general have the potential to improve daily urban activities, providing a series of opportunities concerning urban dimensions such as governance, economy, environment, mobility, people and life. In this perspective, the social, environmental and economic challenges arising from the use of these tools have been explained. The study finds that disruptive urban technologies not only promote highly efficient and computerized urban processes and efficiency, but also improve the understanding of planning, monitoring and analysis of the performance of sensor cities by increasing awareness of citizens, businesses, local authorities, and so on.

The framework of sensor cities (see Figure 2) illustrates and defines the process of detection, collection, processing, analysis of urban data up to their transformation into information necessary for urban policymakers. In this regard, the use of IoT platforms equipped with systems, devices, sensors, algorithms, platforms, models, and so on provides a complete and exhaustive view of the future trends of urban performance measurement methodologies. Furthermore, these methodologies and approaches give rise to some important issues such as data quality and integrity, cyber security, digital data and information ethics, and regulations. These are among the critical issues that prospective research must tackle.

To recap the findings and conclude the paper, we list the following highlights of the study: (a) The proposed framework improves the understanding of the cities’ progress towards becoming sensing localities to address rising issues effectively and efficiently; (b) Achieving a truly smart urbanism requires continuous monitoring trough urban platforms and dashboards; (c) The data-driven approach highlights a multitude of challenges related to urban governance; (d) Urban dashboards improve citizen accountability and awareness on urban issues, and; (e) Data security, quality, integration and stakeholders must be involved in the urban sensor strategy.

## Figures and Tables

**Table 1:** Impact of quality of experience on sensor city, derived from [153].

	Economic, Social and Privacy Implications	E-government	Health and Assisted Living	Intelligent Transportation Systems	Smart Grids, Energy Efficiency, and Environment
Quality of Experience	Usability	Usability	Usability	Usability	Usefulness
	Personalization	Personalization	Availability	Usefulness	Accessibility
	Transparency	Transparency	Personalization	Effectiveness	Personalization
			Effectiveness	Accessibility	
			Accessibility	Efficiency	
			Efficiency		

## Appendix A

**Table A1:** Sensor city projects of high-tech companies investigated in this study (source: authors).

Location		High-Tech Company Partner	Sensor City Project	Focus	Source
City	Nation				
Toronto	Canada	Google	Sidewalk Labs	Building, energy, waste, environment, water	[182]
Hamburg Port	Germany	Cisco	smartROAD	Building, energy, water, mobility	[183]
Gelsenkirchen	Germany	Huawei	Gelsenkirchen: A Small, Smart City with Big Plans	Mobility, environment, energy	[184]
Adelaide	Australia	Cisco	Lighthouse City	Mobility, environment	[185]
Kansas City	U.S.A.	Cisco	Smart+Connected Communities	Mobility, water, energy	[186]
Singapore	Singapore	Google	Smarter Digital City 3.0	Mobility, living, people, finance, retail	[187]
Friedrichshafen	Germany	Deutsche Telekom	T-City Friedrichshafen	Energy, mobility, healthcare, building	[188]
Charlotte	U.S.A.	Microsoft	Charlotte sustainability city	Energy, mobility, education, safety	[189]
Dublin	Ireland	IBM	SMART Dublin	Mobility, environment, building, water, energy	[190,191]
Aspern Seestadt	Austria	Siemens	Aspern Smart City Research	Building, energy, environment, water	[192]
Stockholm Port	Sweden	Ericsson	Stockholm Royal Seaport’s Smart Energy City project	Water, environment, energy	[193]
Dallas	U.S.A.	Ericsson	Factory of the Future	Mobility	[194]
Espoo	Finland	Nokia	Luxturrim5G Project	Environment, mobility, safety, energy	[195]
Hangzhou	China	Alibaba	City Brain	Mobility, water, environment, healthcare	[144]
Maharashtra	India	Oracle	Centre of Excellence	Mobility, water, education	[196]
Bari Matera	Italy	TIM	Bari Matera 5G	Tourism, culture, mobility, safety, environment, healthcare, agriculture	[197]
Saemangeum	South Korea	Samsung	Green Energy Industrial Complex	Energy, education, agriculture, tourism, environment	[198]
Shenzhen	China	Tencent	Tencent Campus	Energy, environment, mobility, education	[199]
Guiyang	China	Alibaba, Tencent, Apple	Guiyang Sunac City	Environment, mobility, recreation	[200]
Valencia	Spain	Telefonica	Valencia Smart City Platform	Mobility, waste, environment, energy	[201]
